# Effects of Annealing Conditions on Mixed Lead Halide Perovskite Solar Cells and Their Thermal Stability Investigation

**DOI:** 10.3390/ma10070837

**Published:** 2017-07-21

**Authors:** Haifeng Yang, Jincheng Zhang, Chunfu Zhang, Jingjing Chang, Zhenhua Lin, Dazheng Chen, He Xi, Yue Hao

**Affiliations:** 1 Wide Bandgap Semiconductor Technology Disciplines State Key Laboratory, School of Microelectronics, Xidian University, Xi’an 710071, China; faircl@163.com (H.Y.); jchzhang@xidian.edu.cn (J.Z.); zhlin@xidian.edu.cn (Z.L.); dzchen@xidian.edu.cn (D.C.); hxi@xidian.edu.cn (H.X.); yhao@xidian.edu.cn (Y.H.); 2 College of Physics and Optoelectronics Technology, Baoji University of Arts and Sciences, Baoji 721016, China; 3 Shaanxi Joint Key Laboratory of Graphene, Xidian University, Xi’an 710071, China

**Keywords:** perovskite solar cells, annealing condition, film quality, thermal stability

## Abstract

In this work, efficient mixed organic cation and mixed halide (MA_0.7_FA_0.3_Pb(I_0.9_Br_0.1_)_3_) perovskite solar cells are demonstrated by optimizing annealing conditions. AFM, XRD and PL measurements show that there is a better perovskite film quality for the annealing condition at 100 °C for 30 min. The corresponding device exhibits an optimized PCE of 16.76% with *V*_OC_ of 1.02 V, *J*_SC_ of 21.55 mA/cm^2^ and FF of 76.27%. More importantly, the mixed lead halide perovskite MA_0.7_FA_0.3_Pb(I_0.9_Br_0.1_)_3_ can significantly increase the thermal stability of perovskite film. After being heated at 80 °C for 24 h, the PCE of the MA_0.7_FA_0.3_Pb(I_0.9_Br_0.1_)_3_ device still remains at 70.00% of its initial value, which is much better than the control MAPbI_3_ device, where only 46.50% of its initial value could be preserved. We also successfully fabricated high-performance flexible mixed lead halide perovskite solar cells based on PEN substrates.

## 1. Introduction

Because of their exciting optoelectronic properties and low-cost solution processes, perovskite solar cells (PSCs) are considered as a favorable candidate for next-generation photovoltaic technology [1,2,3,4]. Over the past few years, the power conversion efficiency (PCE) of PSCs has been rapidly increased from about 4% to over 20% [5,6,7]. Generally, PSCs can be classified into two typical structures: the mesoporous structure and the planar structure [8,9]. Among the planar structure, PSCs with a p-i-n structure using poly (3,4-ethylenedioxythiophene):poly(styrenesulfonate) (PEDOT:PSS) as the hole transport layer and [6,6]-phenyl-C61-butyric acid methyl ester (PCBM) as the electron transport layer have been considered to have a promising structure due to the low-temperature fabrication process, simple preparation methods, lower hysteresis effects and compatibility with flexible substrates [10,11,12].

Organic-inorganic metal halide perovskites adopt the chemical formula ABX_3_, where A is an organic cation (typically MA: CH_3_NH^3+^ or FA:CH(NH_2_)^2+^), B is a metal cation (typically Pb^2+^ or Sn^2+^), and X is a halide anion (typically Cl^−^, Br^−^, or I^−^). By controlling the chemical compositions, many important favorable properties of these perovskites can be achieved [13,14]. Because perovskite containing chlorine (CH_3_NH_3_PbI_3-x_Cl_x_) has a much longer electron-hole diffusion length compared to pure iodine-based perovskite (CH_3_NH_3_PbI_3_) [3], the performance of PSCs based on CH_3_NH_3_PbI_3-x_Cl_x_ has significantly improved [15]. Replacing methylammonium (MA) with formamidinium (FA) can narrow the bandgap of perovskite and enhance its thermal stability [16]. After incorporation of bromine into the perovskite, the bandgap of perovskite could be expanded, and the diffusion length could be increased [17]. Recently, mixed perovskite systems (MA_x_FA_1-x_Pb(I_y_Br_1-y_)_3_) have shown great advantages in terms of PCE [18,19,20,21]. There have been systematic chemical composition studies on the optical properties, crystal structure, surface morphology, and photovoltaic properties of the compositional MA_x_FA_1-x_Pb(I_y_Br_1-y_)_3_ with both mesoporous and planar structures [20,21]. Our team also independently found that the optimal compositional perovskite is MA_0.7_FA_0.3_Pb(I_0.9_Br_0.1_)_3_ in an inverted planar structure, which is in accordance with what other teams have reported [21].

Beside the detailed composition of the perovskite, the annealing conditions for perovskite films also have a great influence on the performance of PSCs. However, the reported annealing conditions varies greatly between MA/FA-Pb-I/Br mixed perovskite systems [18,19,20,21]. For example, annealing times ranging from 10 to 70 min have been reported. More importantly, although the performance of PSCs based on an MA/FA-Pb-I/Br mixed perovskite system has been reported, there has still been little research carried out on the thermal stability of the fabricated MA_0.7_FA_0.3_Pb(I_0.9_Br_0.1_)_3_ PSCs, until now. In this work, the effects of annealing conditions on the performance of PSCs and the corresponding device thermal stability were systematically investigated based on the MA_0.7_FA_0.3_Pb(I_0.9_Br_0.1_)_3_ materials. We found that when the annealing condition was 30 min at 100 °C, the quality of MA_0.7_FA_0.3_Pb(I_0.9_Br_0.1_)_3_ perovskite films on the ITO/PEDOT:PSS substrate were the best, and the corresponding PSC also showed an optimized performance. The best-performing device exhibited a PCE of 16.76% with *V*_OC_ of 1.02 V, *J*_SC_ of 21.55 mA/cm^2^ and FF of 76.27%. More importantly, the film quality investigation and the device characterization show that this mixed lead halide perovskite based on 30% FA cations and 10% bromine anions can significantly increase the thermal stability of perovskite film. In addition, we successfully fabricated high-performance flexible mixed-lead halide perovskite solar cells based on PEN substrates.

## 2. Experimental Section

### 2.1. Materials and Reagents

All solvents and reagents, unless stated otherwise, were of analytically pure quality and used as received. PbI_2_ (beads, 99.999%) and PbBr_2_ (extra pure, 99.999%) were purchased from Alfa Aesar (Ward Hill, MA, USA). Methylammonium iodide (MAI) and Formamidinium iodide (FAI) were purchased from Dyesol (Queanbeyan, New South Wales, Australia). Poly (3,4-ethylenedioxythiophene):poly(styrenesulfonate) (PEDOT:PSS Clevios P VP Al 4083) solution was acquired from Heraeus (Hanau, Hesse, Germany). Phenyl-C61-butyric acid methyl ester (PCBM) was acquired from American Dye Source (Baie d’Urfé, QC, Canada), Bathocuproin (BCP, 98%) was acquired from Alfa Aesar (Ward Hill, MA, USA), and butyrolactone (GBL, ≥99.9%) was purchased from Aladdin (Shanghai, China). Other materials, including dimethyl sulfoxide (DMSO, ≥99.7%), chlorobenzene (anhydrous, 99.8%) and isopropanol (IPA, anhydrous, 99.5%), were supplied by Sigma-Aldrich (St. Louis, MO, USA).

### 2.2. Fabrication of Perovskite Solar Cells

**Figure 1:**
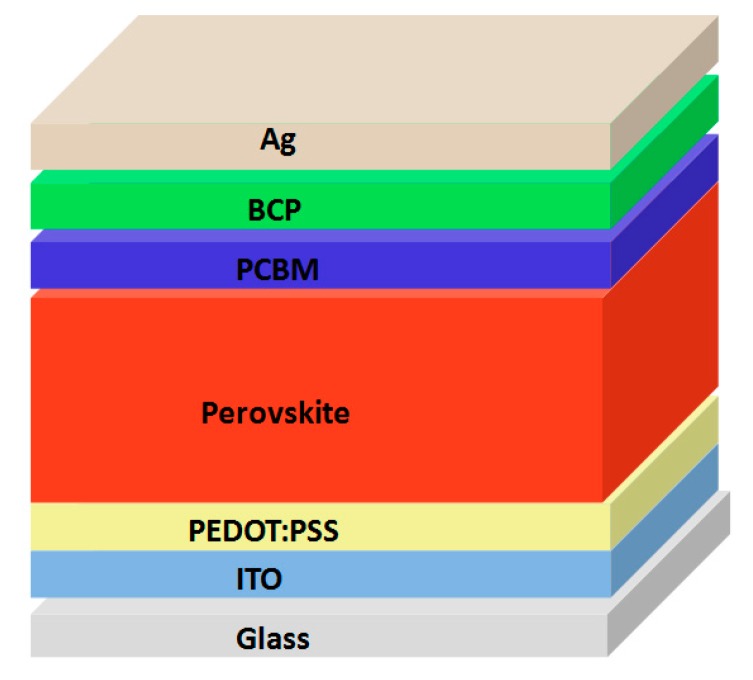
Device structure of p-i-n MA_0.7_FA_0.3_Pb(I_0.9_Br_0.1_)_3_ PSCs.

The structure of the fabricated perovskite solar cells was ITO/PEDOT:PSS/perovskite/PCBM/BCP/Ag, as shown in Figure 1. Planar PSCs were fabricated on pre-patterned ITO glass substrates (10 Ω per square, 2.0 cm × 2.5 cm in size). The ITO glass substrates were sequentially cleaned with 5% Decon-90 solution, de-ionized water, acetone and isopropyl alcohol for 20 min, respectively. They were cleaned in a UV ozone oven for 15 min before the device fabrication. A layer of PEDOT:PSS was spun coated onto the ITO substrate at 7000 rpm for 40 s. Then, they were annealed at 140 °C for 15 min. The substrates were transferred into a glove box filled with nitrogen. MA_0.7_FA_0.3_Pb(I_0.9_Br_0.1_)_3_ perovskite precursors were prepared by dissolving MAI, FAI, PbI_2_ and PbBr_2_ with 0.945 M, 0.405 M, 1.19 M and 0.21 M into co-solvent of DMSO:GBL (3:7 vol. ratio) stirred for 2 h at 70 °C. The ratio of [Pb^2+^]:([MA]+[FA]) for our optimized precursor solution was 1.4:1.35. And MAPbI_3_ perovskite precursors were prepared by dissolving MAI, and PbI_2_ with 1.35 M and 1.4 M into co-solvent of DMSO:GBL (3:7 vol. ratio) stirred for 2 h at 70 °C. The spin-coater was started at a rotation speed of 1000 rpm for 15 s and 5000 rpm for another 25 s. 350 μL toluene was added quickly at 35 s after the start of the spin coating process. The perovskite films were then annealed at 100 °C for 10 min, 20 min, 30 min, and 40 min for MA_0.7_FA_0.3_Pb(I_0.9_Br_0.1_)_3_ films and 20 min for MAPbI_3_ films. The samples for the PL measurement were prepared as the same process of device fabrication on glasses annealed at 100 °C for 10 min, 20 min, 30 min, and 40 min for MA_0.7_FA_0.3_Pb(I_0.9_Br_0.1_)_3_ films. The samples for the thermal stability tests were prepared using the same process of device fabrication, on glasses annealed at 100 °C for 30 min for MA_0.7_FA_0.3_Pb(I_0.9_Br_0.1_)_3_ films, and 20 min for MAPbI_3_ films. 20 mg/mL chlorobenzene solution of PCBM was spin-coated at 2000 rpm for 40 s. The thickness of PCBM film was about 50 nm. The thin layer of BCP (0.5 mg/mL in IPA) was deposited on the top of the PCBM layer at 6000 rpm for 40 s. Finally, the films were transferred to a metal evaporation chamber, and 100 nm thick Ag contacts were deposited under high vacuum (<4 × 10^−4^ Pa). The active area was 0.07 cm^2^, defined by a shadow mask. The tests for the thermal stability of perovskite films and the devices were carried out inside the N_2_ atmosphere glove box (oxygen ≤ 10 ppm; water ≤ 1 ppm) to exclude the effects of moisture and oxygen.

### 2.3. Characterization 

The morphologies of the films were characterized using a JSM-7800F extreme-resolution analytical field emission scanning electron microscope (SEM). The roughness was determined using atomic force microscopy (AFM) (Bruker Dimension Icon, Bruker, Germany). The UV-vis absorption measurements were carried out using a PerkinElmer Lambda 950 UV-vis spectrophotometer. X-ray diffraction (XRD) patterns were obtained using an X-ray diffractometer (D8 Advance, Bruker, Germany) using Cu Kα radiation. The photovoltaic performance of PSCs was measured with a computer-programmed Keithley 2400 source/meter under an AAA solar simulator (XES-301, SEN-EI Electric. Co. Ltd, Osaka, Japan), AM 1.5 G illumination with an intensity of 100 mW/cm^2^ (1 sun, calibrated by a NREL-traceable KG5 filtered silicon reference cell). Incident photo-to-current conversion efficiencies (IPCEs) of perovskite solar cells were measured by the solar cell quantum efficiency measurement system (SCS10-X150, Zolix Instrument. Co. Ltd, Beijing, China).

## 3. Results and Discussion

The device structure of p-i-n PSCs has a configuration of Glass/ITO/PEDOT:PSS/MA_0.7_FA_0.3_Pb(I_0.9_Br_0.1_)_3_/PCBM/BCP/Ag as shown in Figure 1. Because bathocuproine (BCP) possesses deep HOMO energy level (−7.0 eV), it has been employed to act as the hole blocking layer, and to modify the cathode for better electron collection [22,23,24].

In order to achieve highly uniform and smooth perovskite films, we adopted a solvent-engineering technique by employing the anti-solvent dripping methods as the previous report [25]. Because the presence of DMSO helps to retard (or decrease) the reaction between PbI_2_ and MAI components of perovskite by forming DMSO–PbI_2_ complexes, a smoother film is formed upon a consecutive spin-coating process. The role of adding anti-solvent is to uniformly induce a rapid increase of concentration of perovskite precursor materials, forming an intermediate phase by the excess solvent being washed away. The effect of excess PbI_2_ on the properties of perovskite thin films, and the photovoltaic performance and stability of PSCs has been thoroughly researched by other groups and our group [4,20,26,27]. Because the presence of a slight excess of unreacted lead iodide can improve the quality of perovskite films, passivate the perovskite grain boundaries, and suppress the charge carrier recombination, the photovoltaic performance and stability of PSCs can be enhanced. The ratio of [Pb^2+^]:([MA] + [FA]) for our optimized precursor solution was 1.4:1.35.

**Figure 2:**
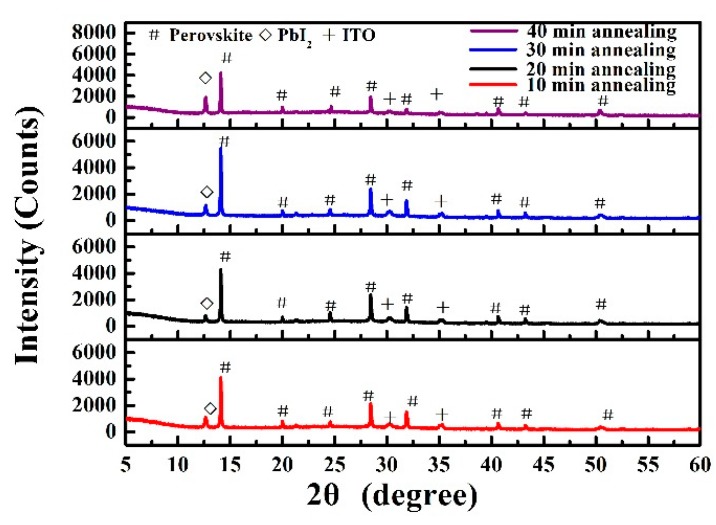
XRD patterns of MA_0.7_FA_0.3_Pb(I_0.9_Br_0.1_)_3_ perovskite films on glass/ITO substrates annealed at 100 °C for 10, 20, 30 or 40 min.

Figure 2 shows the X-ray diffraction (XRD) patterns of MA_0.7_FA_0.3_Pb(I_0.9_Br_0.1_)_3_ perovskite films on glass/ITO annealed at 100 °C for 10, 20, 30 and 40 min, respectively. The main Bragg diffraction peaks at 2θ = 14.1°, 28.4°, 31.8° and 50.2° could correspond to the (110), (220), (310), (044) planes of the perovskite crystalline structure, respectively [20,28,29,30]. Due to the slight excess of lead iodide, there is another strong Bragg diffraction peak at 2θ = 12.6°, which could be attributed to the (006) face of PbI_2_ [31]. As we can see that the main perovskite diffraction peaks are significantly enhanced with the increase of the annealing time from 10 to 30 min, indicating an improvement in crystallinity. However, with a further increase of annealing time to 40 min, the peaks belonging to PbI_2_ increased only slightly, indicating that the perovskite film should be mildly decomposed.

**Figure 3:**
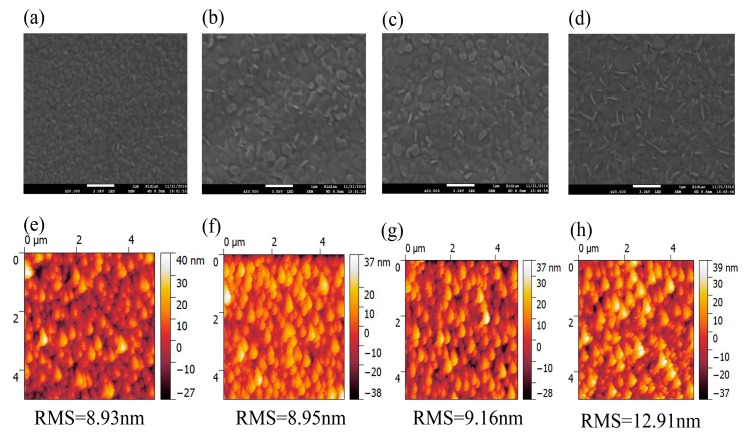
SEM and AFM topography images of MA_0.7_FA_0.3_Pb(I_0.9_Br_0.1_)_3_ perovskite films annealed at 100 °C for 10 (**a**,**e**), 20 (**b**,**f**), 30 (**c**,**g**) and 40 (**d**,**h**) min. The scale bar in SEM measurements is 1 μm.

Figure 3 displays the surface morphologies of MA_0.7_FA_0.3_Pb(I_0.9_Br_0.1_)_3_ perovskite films annealed at 100 °C for 10, 20, 30 and 40 min by the top-view SEM and AFM (AFM phase images are shown in Figure S1). The grain size distribution histograms of MA_0.7_FA_0.3_Pb(I_0.9_Br_0.1_)_3_ perovskite films annealed at 100 °C for 10, 20, 30 and 40 min are shown in Figure S2. As can be seen, the average grain size of MA_0.7_FA_0.3_Pb(I_0.9_Br_0.1_)_3_ film is relatively small when the annealing time is only 10 min (around 150–300 nm), but it becomes larger (around 300–500 nm) when the annealing time is more than 10 min. With the increase of the annealing time, the roughness of MA_0.7_FA_0.3_Pb(I_0.9_Br_0.1_)_3_ films is slightly increased (Figure 3e–h). The root-mean-square (RMS) roughness value of MA_0.7_FA_0.3_Pb(I_0.9_Br_0.1_)_3_ film with the annealing time of 30 min in an area of 5 μm × 5 μm is about 9 nm. However, when the annealing time was prolonged to 40 min, the RMS roughness value of perovskite film obviously increased to about 13 nm. The rougher morphology of perovskite film and the larger contrast in AFM phase images with the annealing time above 40 min indicate that the perovskite film has been slightly decomposed to PbI_2_ [32], and this is consistent with the XRD results.

**Figure 4:**
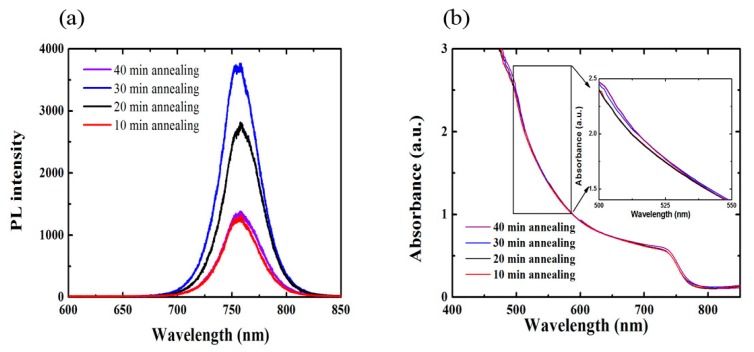
(**a**) Steady-state photoluminescence (PL) spectra for the MA_0.7_FA_0.3_Pb(I_0.9_Br_0.1_)_3_ perovskite films on glass substrates at 100 °C for 10, 20, 30 and 40 min; (**b**) The wavelength-dependent absorbance spectra of MA_0.7_FA_0.3_Pb(I_0.9_Br_0.1_)_3_ perovskite films on glass/ITO annealed at 100 °C for 10, 20, 30 and 40 min. The inset picture is high magnification.

For steady-state PL spectra measurements, all perovskite films were prepared on glass substrates to avoid quenching at the interfaces. All the thin films show a PL emission peak around 758 nm. The PL intensity of perovskite film annealed for 30 min is stronger than other samples (as shown in Figure 4a). It means that the perovskite film annealed at 100 °C for 30 min achieves a film with fewer defects related to the non-radiative recombination centers, which is consistent with the results from the XRD and AFM measurements. The wavelength-dependent absorbance spectra of MA_0.7_FA_0.3_Pb(I_0.9_Br_0.1_)_3_ perovskite films on glass/ITO substrates annealed at 100 °C for 10, 20, 30 and 40 min are shown in Figure 4b. The differences in absorbance spectra among these films are very small. By increasing the duration of the annealing time, the absorbance of the MA_0.7_FA_0.3_Pb(I_0.9_Br_0.1_)_3_ perovskite films become slightly stronger (inset of Figure 4b).

**Figure 5:**
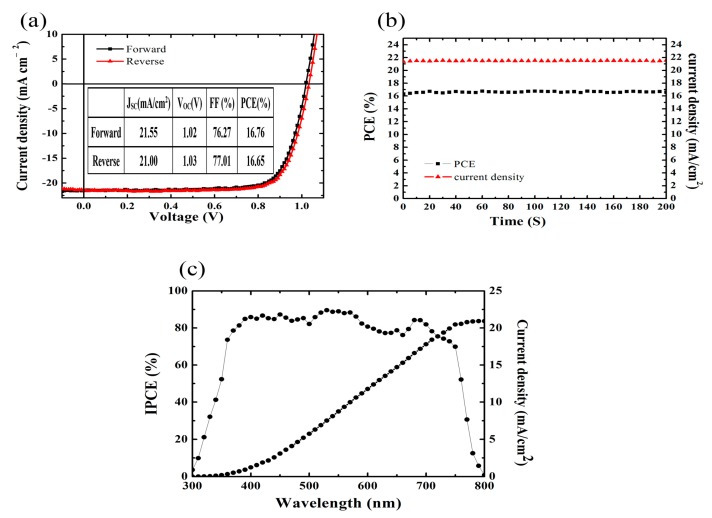
(**a**) *J*-*V* characteristics of forward and reverse bias sweeps for the best-performing MA_0.7_FA_0.3_Pb(I_0.9_Br_0.1_)_3_ PSC annealed at 100 °C for 30 min; (**b**) Steady measurement at the maximum power output point; (**c**) IPCE spectrum of the best-performing solar cell.

The key average *J*-*V* parameters with the different annealing time at 100 °C under AM 1.5 G illumination, including *V*_OC_, *J*_SC_, FF and PCE, are listed in Table 1. With the increase of annealing time, *V*_OC_ remains almost unchanged. Because of the gradual improvement of crystallinity and perovskite content by extending annealing time from 10 to 30 min, *J*_SC_ and FF obviously increased. For the devices with 30 min annealing time, the PCE was improved to 15.70 ± 0.62% with *J*_SC_ of 20.12 ± 0.91 mA/cm^2^ and FF of 78.67 ± 2.93%. The best device shows a PCE of 16.76% with *V*_OC_ of 1.02 V, *J*_SC_ of 21.55 mA/cm^2^ and FF of 76.27%. As shown in Figure 5a, the device exhibits less photocurrent hysteresis with different scanning directions. The steady photocurrent and the stabilized PCE at the maximum power output point (0.84 V) are shown in Figure 5b, and consistent with those from *J*-*V* measurements, indicating that the devices have reliable output. The incident photo-to-electron conversion efficiency (IPCE) curve of the best-performing device is shown in Figure 5c. The integrated current density from the IPCE curve is 20.93 mA/cm^2^, which is in close agreement with the *J*_SC_ measured under the simulated sunlight. The mismatch between the integrated *J*_SC_ obtained from the IPCE curve and the *J*_SC_ obtained from the *J-V* curve is within 3%. Because the MA_0.7_FA_0.3_Pb(I_0.9_Br_0.1_)_3_ perovskite film is slightly decomposed as the annealing time is extended to 40 min, the PCE decreases to 13.53 ± 0.71% with the *J*_SC_ of 18.17 ± 0.74 mA/cm^2^ and FF of 77.52 ± 5.17%.

We also investigated the effect of annealing temperature on the performance of PSCs as fixing the annealing time at 30 min. The key average *J*-*V* parameters with different annealing temperature at 90 °C, 100 °C and 110 °C for the annealing time at 30 min under AM 1.5 G illumination are listed in Table S1. It is shown that when the annealing temperature is below or above 100 °C for 30 min, the photovoltaic performance will deteriorate. Hence, the best annealing conditions for MA_0.7_FA_0.3_Pb(I_0.9_Br_0.1_)_3_ perovskite solar cells is at 100 °C for 30 min.

**Figure 6:**
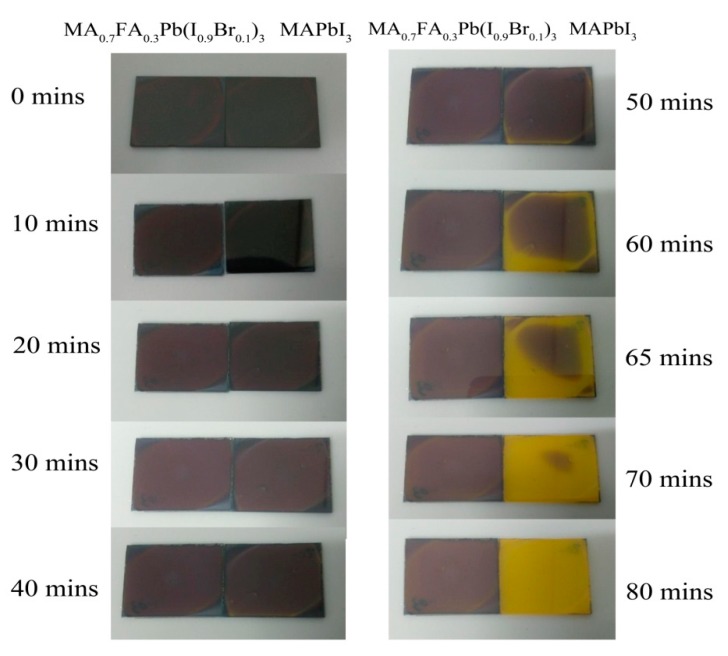
Thermal degradation of MA_0.7_FA_0.3_Pb(I_0.9_Br_0.1_)_3_ and MAPbI_3_ films on glass, when each perovskite is heated at 150 °C for the times indicated.

It was reported that when MA cations were replaced by FA cations, the thermal stability of perovskite solar cells significantly increased [16]. In our study, we found that the mixed lead halide perovskite (MA_0.7_FA_0.3_Pb(I_0.9_Br_0.1_)_3_) based on 30% FA cations and 10% bromine anions obviously increased the stability of the perovskite film at high temperature, and could therefore increase the thermal stability of PSCs. It needs to be mentioned that after being stored at 150 °C for 50 min, the perovskite film based on the pure MA cations (MAPbI_3_) evidently turned yellow in color, while the mixed lead halide perovskite (MA_0.7_FA_0.3_Pb(I_0.9_Br_0.1_)_3_) film remains its initial brown color even after being stored at 150 °C for 80 min (Figure 6).

**Figure 7:**
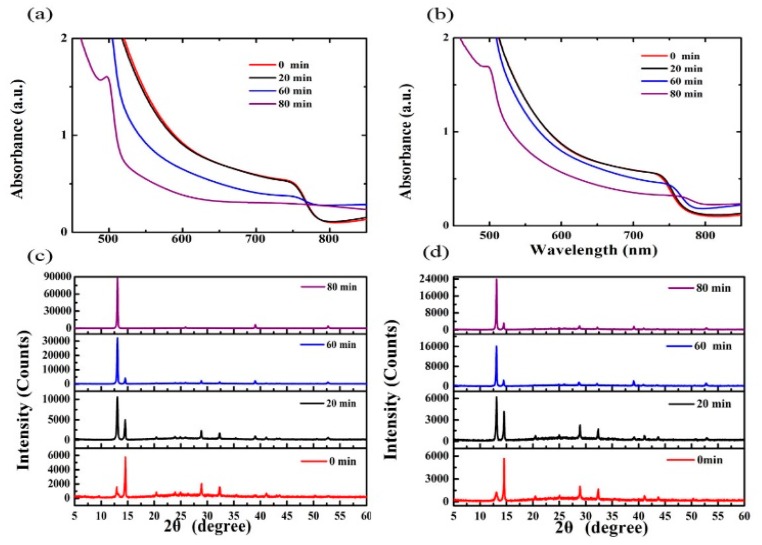
(**a**) The wavelength-dependent absorbance spectra of MAPbI_3_ films; (**b**) MA_0.7_FA_0.3_Pb(I_0.9_Br_0.1_)_3_ films; (**c**) XRD patterns of MAPbI_3_ films; and (**d**) MA_0.7_FA_0.3_Pb(I_0.9_Br_0.1_)_3_ films on glass at 150 °C for the different time scales.

We investigated the UV–vis spectra and XRD patterns of MAPbI_3_ films and MA_0.7_FA_0.3_Pb(I_0.9_Br_0.1_)_3_ films stored at 150 °C for some key time scales (Figure 7). The red curves in Figure 7a,b shows the UV-vis absorption spectra of the initial MAPbI_3_ and MA_0.7_FA_0.3_Pb(I_0.9_Br_0.1_)_3_ films. The absorption onsets of these perovskite films are at about 790 nm, which means that they have a similar optical bandgap of 1.57 eV. As time went on at 150 °C, the absorbance of the MAPbI_3_ films decreased more dramatically than the MA_0.7_FA_0.3_Pb(I_0.9_Br_0.1_)_3_ films. In particular, the onset of perovskite at 790 nm disappeared for the MAPbI_3_ films after being stored at 150 °C for 80 min. There was an obvious absorption onset at about 790 nm for the MA_0.7_FA_0.3_Pb(I_0.9_Br_0.1_)_3_ films after being stored at 150 °C for 80 min. For the same time points at 150 °C, the main diffraction peaks of the MAPbI_3_ films belonging to PbI_2_ (2θ = 12.6°) were obviously stronger than those of the MA_0.7_FA_0.3_Pb(I_0.9_Br_0.1_)_3_ films. After being stored at 150 °C for 80 min, the main perovskite diffraction peaks (2θ = 14.1°) can been still seen for the MA_0.7_FA_0.3_Pb(I_0.9_Br_0.1_)_3_, and their counterparts disappeared for MAPbI_3_ films at 80 min. The XRD results also reveal that the MAPbI_3_ films decomposed faster at high temperature than the MA_0.7_FA_0.3_Pb(I_0.9_Br_0.1_)_3_ films. This shows that the MA_0.7_FA_0.3_Pb(I_0.9_Br_0.1_)_3_ film has a better thermal stability.

We also tested *J-V* parameters before and after heated at 80 °C for 24 h for these two types of PSCs. As shown in Table 2, the PCE had significantly decreased to 46.5% for the MAPbI_3_, but the PCE of the MA_0.7_FA_0.3_Pb(I_0.9_Br_0.1_)_3_ device remained 70.0% of original value.

**Figure 8:**
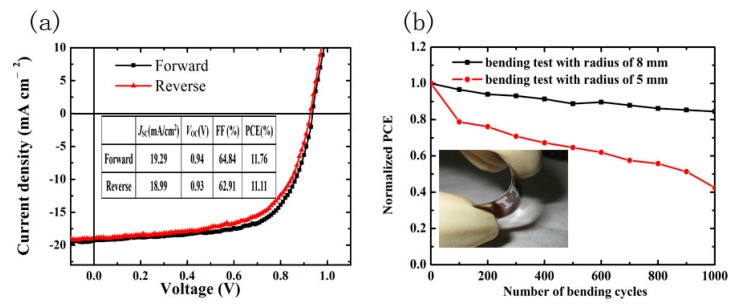
(**a**) *J*-*V* characteristics of forward and reverse bias sweeps for the best-performing flexible PSCs; (**b**) Normalized PCE of flexible perovskite devices as a function of bending cycles with different radii of 8 and 5 mm. Inset is a photograph of the flexible device.

Finally, in order to achieve a flexible device, high-performance mixed-lead halide (MA_0.7_FA_0.3_Pb(I_0.9_Br_0.1_)_3_) PSCs based on flexible PEN substrates were also fabricated by optimizing the perovskite film annealing condition to 30 min. The statistics of PCE, *V*oc, *J*sc and FF distribution of 10 flexible devices from the same batch are shown in Figure S3, and demonstrate that the flexible devices have good reproducibility. The best performance flexible solar cell exhibits *V*_OC_ of 0.94 V, *J*_SC_ of 19.29 mA/cm^2^, FF of 64.83%, and PCE of 11.76%. For the reverse bias, the device exhibits *V*_OC_ of 0.93 V, *J*_SC_ of 18.99 mA/cm^2^, FF of 62.91%, and PCE of 11.11%, showing less photocurrent hysteresis with different scanning directions, as shown in Figure 8a.

To quantitatively evaluate the mechanical robustness of our flexible PSCs based on mixed-lead halide, each device’s performance was measured through multiple cycles of bending tests with different bending radii of 8 and 5 mm. Normalized PCEs of flexible devices as a function of the number of bending cycles are illustrated in Figure 8b. The device retains about 85% of its initial PCE after 1000 bending cycles under a bending radius of 8 mm. These results show that the mixed-lead halide (MA_0.7_FA0_.3_Pb(I_0.9_Br_0.1_)_3_) flexible PSCs possess good mechanical robustness, similar to the performance of CH_3_NH_3_PbI_3−x_Cl_x_ flexible PSCs [33]. However, under a serious bending radius of 5 mm, the device performance is degraded dramatically. We think the main reason for the degradation of performance under a bending radius of 5 mm is due to the limitations of the mechanical properties of the flexible substrate, as our team has previously reported with regard to flexible organic solar cells [34].

## 4. Conclusions

In summary, we have demonstrated efficient mixed organic cation and mixed halide (MA_0.7_FA_0.3_Pb(I_0.9_Br_0.1_)_3_) PSCs by optimizing the perovskite film annealing time at 100 °C for 30 min. AFM, XRD and PL measurements show that there is better perovskite film quality under such annealing conditions. The best-performing device exhibits PCE of 16.76% with *V*_OC_ of 1.02 V, *J*_SC_ of 21.55 mA/cm^2^ and FF of 76.27%. More important, the mixed-lead halide perovskite based on 30% FA cations and 10% bromine anions can significantly increase the thermal stability of perovskite film. After being heated at 80 °C for 24 h, it is shown that the PCE is significantly decreased to 46.50% of its initial value for the MAPbI_3_ device, but the PCE of the MA_0.7_FA_0.3_Pb(I_0.9_Br_0.1_)_3_ device remains 70.00% of original value. We also successfully fabricated high-performance flexible mixed-lead halide perovskite solar cells based on PEN substrates.

## Figures and Tables

**Table 1:** Photovoltaic parameters of MA_0.7_FA_0.3_Pb(I_0.9_Br_0.1_)_3_ perovskite solar cells annealed at 100 °C for 10, 20, 30 and 40 min. ^a^

Annealing Time (min)	*J*_SC_ (mA/cm^2^)	*V*_OC_ (V)	FF (%)	PCE (%)	Best PCE (%)
10	17.84 ± 1.27	1.02 ± 0.01	69.92 ± 4.09	12.65 ± 0.50	13.30
20	18.01 ± 0.69	0.98 ± 0.01	78.41 ± 1.18	13.83 ± 0.73	14.63
30	20.12 ± 0.91	0.99 ± 0.03	78.67 ± 2.93	15.70 ± 0.62	16.76
40	18.17 ± 0.74	0.96 ± 0.01	77.52 ± 5.17	13.53 ± 0.71	14.30

^a^ Each value is derived from 10 cells made from two separate batches.

**Table 2:** Photovoltaic parameters of MAPbI_3_and MA_0.7_FA_0.3_Pb(I_0.9_Br_0.1_)_3_ PSCs before and after 24 h heated at 80 °C. ^a^

Devices		*J*_SC_ (mA/cm^2^)	*V*_OC_ (V)	FF (%)	PCE (%)
MAPbI_3_ Solar cells	initial	19.30 ± 0.25	0.99 ± 0.01	73.33 ± 0.64	13.96 ± 0.17
	24 h heated at 80 °C	12.56 ± 1.09	0.98 ± 0.02	53.15 ± 6.34	6.50 ± 0.17
MA_0.7_FA_0.3_Pb(I_0.9_Br_0.1_)_3_ Solar Cells	initial	20.10 ± 0.95	1.00 ± 0.03	78.86 ± 2.49	15.76 ± 0.63
	24 h heated at 80 °C	16.40 ± 0.48	0.98 ± 0.01	68.68 ± 1.88	11.03 ± 0.15

^a^ Each value is derived from 5 cells.

## Supplementary Materials

The following are available online at www.mdpi.com/1996-1944/10/7/837/s1, Figure S1: AFM phase images of MA_0.7_FA_0.3_Pb(I_0.9_Br_0.1_)_3_ perovskite films annealed at 100 °C for 10 (a), 20 (b), 30 (c) and 40 (d) min, Figure S2: SEM images and grain size distribution histograms of MA_0.7_FA_0.3_Pb(I_0.9_Br_0.1_)_3_ perovskite films annealed at 100 °C for 10 (a), 20 (b), 30 (c) and 40 (d) min. Image size was 2.2 μm × 2.2 μm (The grain size was collected by software “Nano Measurer”), Figure S3: Statistics of PCE (a), *J*_SC_ (b), *V*_OC_ (c) and FF (d) distribution of 10 flexible devices based on 10 devices from the same batch, Table S1: Photovoltaic parameters of MA_0.7_FA_0.3_Pb(I_0.9_Br_0.1_)_3_ perovskite solar cells annealed at 90 °C,100 °C and 110 °C for 30 min.
