# Supplementary material for: Effects of Annealing Conditions on Mixed Lead Halide Perovskite Solar Cells and Their Thermal Stability Investigation
Source: Materials (Basel). 2017 Jul 21;10(7):837. doi: 10.3390/ma10070837 (PMC5551880; doi:10.3390/ma10070837)
Supplement: Supplementary file 1 [file materials-10-00837-s001.pdf]

materials

Supplementary Materials

Effects of Annealing Conditions on Mixed Lead Halide Perovskite Solar Cells and Their Thermal Stability Investigation

Haifeng Yang [1,2], Jincheng Zhang [1,3], Chunfu Zhang [1,3,*], Jingjing Chang [1,*], Zhenhua Lin[1,3], Dazheng Chen [1], He Xi [1] and Yue Hao [1]

[1] Wide Bandgap Semiconductor Technology Disciplines State Key Laboratory, School of Microelectronics, Xidian University, Xi'an 710071, China; faircl@163.com (H.Y.); jchzhang@xidian.edu.cn (J.Z.); zhlin@xidian.edu.cn (Z.L.); dzchen@xidian.edu.cn (D.C.); hxi@xidian.edu.cn (H.X.); yhao@xidian.edu.cn (Y.H.)
[2] College of Physics and Optoelectronics Technology, Baoji University of Arts and Sciences, Baoji 721016
[3] Shaanxi Joint Key Laboratory of Graphene, Xidian University, Xi'an 710071, China
* Correspondence: cfzhang@xidian.edu.cn (C.Z.); jjingchang@xidian.edu.cn (J.C.)

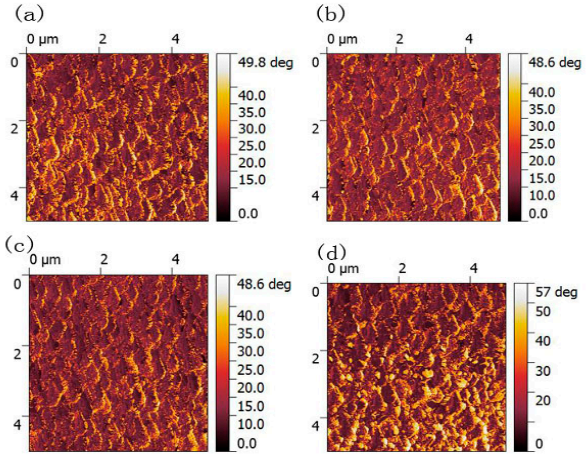

Figure S1. AFM phase images of $MA_{0.7}FA_{0.3}Pb(I_{0.9}Br_{0.1})_3$ perovskite films annealed at 100 °C for 10 (**a**), 20 (**b**), 30 (**c**) and 40 (**d**) min.

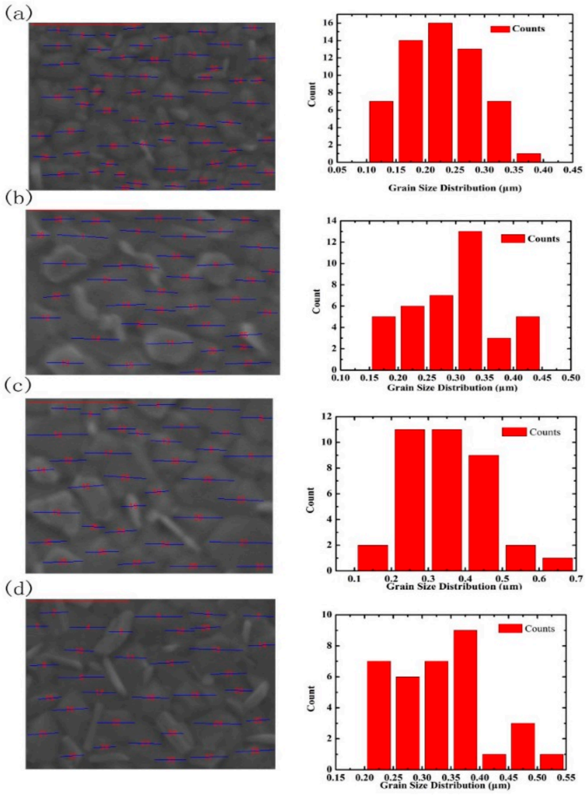

Figure S2. SEM images and grain size distribution histograms of $MA_{0.7}FA_{0.3}Pb(I_{0.9}Br_{0.1})_3$ perovskite films annealed at 100 °C for 10 (**a**), 20 (**b**), 30 (**c**) and 40 (**d**) minutes. Image size was 2.2 μm × 2.2 μm. (The grain size was collected by software "Nano Measurer".)

Table S1. Photovoltaic parameters of $MA_{0.7}FA_{0.3}Pb(I_{0.9}Br_{0.1})_3$ perovskite solar cells annealed at 90 °C,100 °C and 110 °C for 30 min.[a]

Annealing Temperature (°C)	J_{SC} (mA/cm²)	V_{OC} (V)	FF (%)	PCE (%)
90	17.01 ± 0.38	0.96 ± 0.10	77.88 ± 4.42	12.74 ± 0.93
100	19.65 ± 0.46	0.98 ± 0.09	82.43 ± 3.26	15.81 ± 0.58
110	16.97 ± 0.74	1.02 ± 0.14	73.03 ± 5.41	12.59 ± 1.11

[a] Each value is derived from 5 cells made from the same batch.

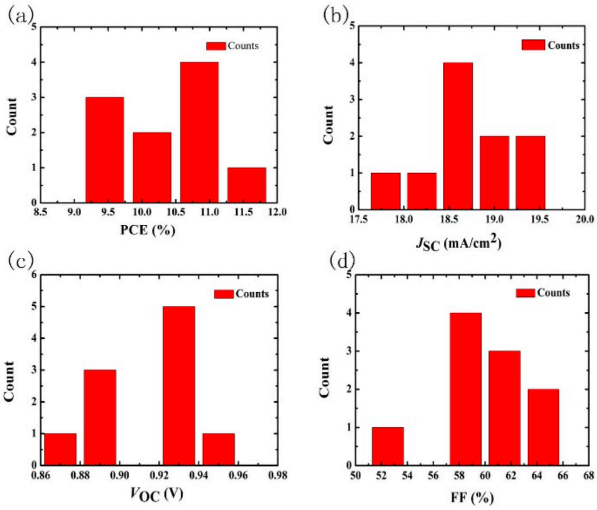

Figure S3. Statistics of PCE (**a**), J_{SC} (**b**), V_{OC} (**c**) and FF (**d**) distribution of 10 flexible devices based on 10 devices from the same batch.
